# Dispositional Awe Predicts Mental Health Through Interpretation Bias During COVID‐19 Transmission: A Longitudinal Study

**DOI:** 10.1002/pchj.70008

**Published:** 2025-03-10

**Authors:** Xiaohan Wang, Li Luo, Jiajin Yuan

**Affiliations:** ^1^ Sichuan Key Laboratory of Psychology and Behavior of Discipline Inspection and Supervision, Institute of Brain and Psychological Sciences Sichuan Normal University Chengdu China; ^2^ The Department of Education Science Neijiang Normal University Neijiang China

**Keywords:** dispositional awe, interpretation bias, longitudinal study, mediation effect, mental health

## Abstract

During a public health emergency, such as the widespread transmission of COVID‐19 following loosened COVID‐19 policies in China, people's mental health is impacted along with their physical well‐being. In order to investigate ways to mitigate these negative effects, this study examined how dispositional awe can predict mental health outcomes during such emergencies using a three‐wave longitudinal design. Five hundred twenty seven participants (mean age = 21.18, SD = 3.39; 368 males) took part in the study within the first 2 months after the implementation of loosened COVID‐19 policies, with one‐month intervals between waves. Cross‐lagged analysis revealed that dispositional awe in Wave 1 significantly predicted higher positive and lower negative interpretation bias in Wave 2, which in turn promoted positive mental functioning in Wave 3. Furthermore, negative interpretation bias in Wave 2 acted as a mediator for the predictive role of dispositional awe in Wave 1 on psychosomatic symptoms in Wave 3. These findings suggest that dispositional awe can act as a protective factor for mental health during the COVID‐19 pandemic by influencing people's interpretation orientation.

## 1 Introduction

Public emergencies, such as public health emergencies, natural disasters, and public safety emergencies, have brought risks to both the physical and mental health of human beings. COVID‐19 pandemic is a typical public health emergency that brought people a high level of depression, anxiety, as well as post‐traumatic stress disorder during the pandemic (Giuntella et al. 2021; Goldrick‐Rab et al. 2022; Lu et al. 2021; Proto and Zhang 2021; Witteveen and Velthorst 2020; Xiong et al. 2020). People also have poor sleep quality (Giuntella et al. 2021; Teague et al. 2022), tense interpersonal relationships (Chakraborty et al. 2021), feelings of social isolation (Lu et al. 2021; Teague et al. 2022), and substance abuse (Tsai et al. 2021). As the threat of the virus weakened, many countries began to adjust their strict pandemic control policies to more loosened ones. Particularly, China implemented loosened policies on December 18, 2022, which resulted in a surge in COVID‐19 infections. This sudden increase led to a significant threat to public mental health during that period. Therefore, it is necessary to study the protective factors for people's mental health.

Prior studies found that excessive self‐centeredness is a major risk factor for mental health problems (Boehme et al. 2015; McGregor et al. 2013; Montoro et al. 2022). Conversely, awe, a self‐transcendent emotion triggered by vast and powerful stimuli that are beyond individuals' understanding (Keltner and Haidt 2003), can shift one's attention to the outside world to decrease self‐centeredness (Shiota et al. 2014; Stellar 2021). A recent study revealed that awe induction reduced the self‐face processing advantage, shown by the lower response accuracy for self‐face than for friend‐face (Zhang 2022). A functional neuroimaging study also suggests that the feeling of awe can decrease activity in the default mode network, a neural network primarily involved in self‐referential processing (van Elk et al. 2019). Schneider (2009) believes that the experience of awe may serve as an important promoter for public mental health. In line with this view, Bernstein and Patrick (2020) found that dispositional awe was positively linked to well‐being. Laboratory research also yielded similar results, showing that individuals experiencing awe were more satisfied with their current lives and thus had greater momentary well‐being, compared with those experiencing other positive or neutral emotions (Gordon et al. 2017; Rudd et al. 2012). In the field of psychotherapy, transpersonal psychotherapy guides clients to experience awe, which can enhance the growth of spirituality and relieve psychological symptoms (Bonner and Edward 2016; Luo et al. 2021). Also, it was indicated that older adults increased their daily prosocial positive emotions and reduced daily distress after outdoor awe‐inspiring walks (Sturm et al. 2022). Based on this evidence, it is reasonable to infer that dispositional awe may serve as a buffer for people's mental health threats after the release of loosened COVID‐19 policies. However, this proposition needs to be directly tested.

Research on the relationship between awe and mental health has been well‐established. However, there has been limited research focusing on the mechanisms behind this impact. We propose that dispositional awe may elevate mental health via shaping interpretation bias, which refers to the tendency to interpret ambiguous information in a specific way, including positive interpretation bias (e.g., “the person smiling in the audience thinks I am doing a good job”), negative interpretation bias (e.g., “the person smiling in the audience is mocking me”), and neutral interpretation bias (e.g., “the person smiling in the audience is telling jokes”) (Moser et al. 2012). The theory of mood‐congruent bias posits that individuals tend to maintain consistency between their emotions and cognition, that is, people experiencing a positive or negative emotion would show a cognitive processing advantage for stimuli with the same valence (Voelkle et al. 2014). As a positive self‐transcendent emotion (Bai et al. 2017; Gordon et al. 2017; Stellar et al. 2017), awe has the power to encourage individuals to maintain an open attitude toward experiences, even in the face of difficulty or uncertainty (Büssing et al. 2013; Rankin et al. 2020; Sawada and Nomura 2020; Silvia et al. 2015; Valdesolo and Graham 2014). It also encourages them to focus on the positive aspects of their lives (Schneider 2017, 2009). In addition, indirect evidences also lent support for the relationship between awe and interpretation bias. On the one hand, a prior study discovered that patients with religious belief, an important elicitor to induce the experience of awe (Keltner and Haidt 2003), had higher scores on positive interpretation of their illnesses, compared to non‐religious people (Büssing et al. 2013). On the other hand, gratitude, a positive self‐transcendent emotion similar to awe, can improve people's optimistic interpretation (Yang 2016). Therefore, we hypothesize that dispositional awe is linked with interpretation bias.

The cognitive theory of mental health suggests that interpretation bias plays a significant role in mental health and the development of symptoms (Beck 2008; Hughes and Kendall 2008; Leung et al. 2022). Consistent with this theory, a growing body of empirical evidence has shown a significant influence of interpretation bias on mental health. Positive interpretation bias usually increases positive emotions and life satisfaction and decreases negative emotions and psychosomatic symptoms (Gawęda et al. 2015; Jopling et al. 2021; Rude et al. 2003; Sonoda 2007; Vinograd et al. 2020). Intervention studies have also found that interpretation training can significantly encourage positive interpretation and decrease negative interpretation for ambiguous social situations, which then reduced anxiety and depression while increasing happiness (Hirsch et al. 2018; Mobini et al. 2014; Nejati et al. 2019). Therefore, we assume that interpretation bias may subserve the promoting effects of awe on mental health.

The present study is supposed to examine the predicting effect of dispositional awe on people's mental health, and further explore the mechanisms underlying this association from the perspective of interpretation bias after China's administration of loosened COVID‐19 policies. We hypothesize that dispositional awe may positively predict positive mental functions assessed by life satisfaction and happiness and negatively predict psychosomatic symptoms assessed by SCL‐90. These relationships are mediated by the interpretation bias. Experts predicted a spike in the number of cases within the first 2 months after the implementation of loosened COVID‐19 policies. To better examine the relationship between variables, we collected three‐wave longitudinal data, and the interval between two consecutive waves was 1 month. The specific time frames for the first, second, and third waves were as follows: from December 17 to December 20, 2022; from January 18 to January 21, 2023; and from February 18 to February 19, 2023.

Cross‐lagged analysis was conducted to explore the relationship among dispositional awe, interpretation bias, and mental health measured by positive mental function and psychosomatic symptoms. This study could expand our understanding of how the feeling of awe predicts mental health under COVID‐19 transmission. The internal ethic committee of Sichuan Normal University reviewed and approved this study involving human participants (Protocol number: 20220001). All data have been made publicly available via The Open Science Framework repository, with the name “data about awe and mental health”, and can be accessed at https://osf.io/d7hev/?view_only=a99180e50b734aef99c9843d1c82e753.

## 2 Methods

### 2.1 Participants

Participants were recruited from two high schools in Chengdu and Neijiang in China, and they were encouraged to invite their classmates or families to participate through the snowball sampling method. Participants provided their informed consent to participate, and they could receive ¥40 (approximately $5.76) after completing the questionnaires. To ensure the validity of the data, we informed participants in advance that the payment would be made after the researchers confirmed the quality of the data.

The Monte Carlo Power Analysis for Indirect Effects Application was used to determine the sample size (Schoemann et al. 2017). At least 280 participants are needed to reach a power of 0.80. Five hundred twenty‐eight participants participated in this study. One data point was removed from further analysis because the scores on mental health were over three standard deviations. Therefore, the data of 527 participants were used for analysis (*n*_female_ = 368). Participants' age ranged from 16 to 72, with an average age of 21.18 ± 3.39 years. There were 153 (29.03%) participants from urban areas and 373 (70.97%) from rural areas. The educational level ranged from junior middle school or below to graduate.

### 2.2 Materials

#### 2.2.1 Dispositional Awe

Dispositional awe was measured by the awe sub‐scale in the Dispositional Positive Emotion Scale (DPES‐awe) (Shiota et al. 2006). DPES‐awe has six items, such as “I often feel awe”. Participants responded to each item using a scale ranging from 1 (*strongly disagree*) to 7 (*strongly agree*). The higher score indicates individuals experiencing awe more likely in their daily life. Cronbach's alphas in this study were between 0.83 and 0.85.

#### 2.2.2 Interpretation Bias

The Interpretation Bias Questionnaire (IBQ) was used to assess individuals' interpretation bias of ambiguous scenarios (Amin et al. 1998; Yang 2011). The questionnaire is composed of 22 ambiguous scenarios, such as “You see a group of friends having lunch, they stop talking when you approach.” Participants were requested to read each scenario carefully and envision themselves within it. Each scenario has three possible interpretations, such as “They are about to ask you to join” (positive interpretation), “They were saying negative things about you” (negative interpretation), and “They just ended their conversation” (neutral interpretation), which were randomly presented. Participants were asked to indicate the extent to which they might choose each explanation to interpret the scenario, ranging from 1 (*extremely unlikely*) to 5 (*extremely likely*). Items from each type of interpretation were summed and divided by the total number of responses on that interpretation. The higher the score, people are more inclined to adopt this type of explanation to explain ambiguous situations. Cronbach's alpha for positive, negative, and neutral interpretations in the current study were between 0.84 to 0.89, 0.91 to 0.94, and 0.79 to 87, respectively.

#### 2.2.3 Mental Health

To comprehensively assess mental health, we selected multiple measures, including the Satisfaction with Life Scale (SWLS), the Short Depression–Happiness Scale (SDHS), and the Symptom Checklist 90 (SCL‐90).

SWLS was employed due to that we want to aim to capture the positive subjective experience and overall psychological adjustment of the participants. It is a key element of mental health and well‐being (Diener et al. 1985). SWLS (Diener et al. 1985) is a unidimensional scale, including five items (e.g., “In most ways my life is close to my ideal”). Participants should rate their agreement to each item on a 7‐point scale ranging from 1 (*strongly disagree*) to 7 (*strongly agree*). The higher score indicates greater satisfaction with one's life. Cronbach's alphas were between 0.87 to 0.88 in this study.

SCL‐90 was included because of its wide usage and comprehensiveness in assessing psychological symptoms and distress in both psychiatric and non‐clinical populations (Derogatis et al. 1976). This inventory comprises 90 items and 10 subscales, including anxiety, depression, somatization, phobic anxiety, obsessive‐compulsive, paranoid ideation, interpersonal sensitivity, hostility, psychoticism, and others. Participants were required to rate each item on a 5‐point scale ranging from 0 (*not at all*) to 4 (*extremely*). The total score is used as the index of psychosomatic symptoms. A higher score indicates more severe psychopathology. The Cronbach's alphas for this inventory were 0.99 across all three waves.

SDHS was chosen for its ability to simultaneously measure depressive symptoms and happiness levels, which provides a concise yet effective way to understand emotional well‐being (Joseph et al. 2004). It is unidimensional and consists of six items (e.g., “I felt pleased with the way I am”). Participants should respond to each item on a 4‐point scale ranging from 1 (*never*) to 4 (*often*). Higher score indicates the higher happiness. Cronbach's alphas of this scale were between 0.77 to 0.82 in this study.

By using these three measures in combination, we can construct a more comprehensive and multi‐faceted understanding of the participants' mental health and improve the convergent validity of this research. We divided the indicators of mental health into two dimensions, namely positive and negative. The positive indicators include life satisfaction and happiness, respectively measured by SWLS and SDHS, while the negative indicators cover the psychological symptoms measured by SCL‐90.

#### 2.2.4 Gratitude Questionaires

Gratitude is a self‐transcendent emotion, similar to the feeling of awe (Stellar et al. 2017), which flows from the perception that one has benefited from the costly, or intentional action of another person (McCullough et al. 2001). Considering that gratitude, as an important influence factor for mental health, is often generated together with awe (Wood et al. 2010), we measured gratitude using the Gratitude Questionaires (GQ‐6) (McCullough et al. 2002). This single‐factor scale consists of six items (e.g., “If I had to list everything that I felt grateful for, it would be a very long list.”). Participants should respond to each item on a 7‐point scale ranging from 1 (*strongly disagree*) to 7 (*strongly agree*). The higher score indicates greater tendency to experience the feeling of gratitude. Cronbach's alpha was 0.76 in this study.

In each wave, participants completed DPES‐awe, IBQ, SWLS, SDHS, and SCL‐90. GQ‐6 was administered at Wave 3. We collected additional information as the controlled variables, including age, gender (1 = male, 2 = female), education level (1 = junior middle school or below, 5 = graduate), the number of times they had contacted COVID‐19 (coded as 0—*never*, 1—*once*, 2—*twice*, 3—*more than three times*; *n*(0) = 103), and the severity (1—*not at all severe*, 7—*extremely severe*; *M =* 3.46, SD = 1.49) at Wave 3.

### 2.3 Statistical Analysis

Descriptive statistics analysis was conducted in SPSS 23.0. Cross‐lagged structural equation analysis was estimated in AMOS 23.0 (SPSS Inc., Chicago, IL, USA). In detail, we took the following steps in the data analysis. Firstly, we identified outliers using standard deviation (SD) and examined the descriptive statistics, including the mean and SD of all variables. Secondly, we conducted a correlative analysis to primarily investigate the relationships between variables. Finally, we adopted the Cross‐Lagged Panel Model (CLPM) to conduct Structural Equation Modeling to deeply explore the complex relationships among awe, interpretive bias, and mental health in the longitudinal dimension in AMOS 23.0. We established a second‐order Cross‐Lagged Panel Model (CLPM). Specifically, we constructed the correlations among variables at the same time point and the autoregressive paths of variables. Moreover, we respectively constructed the predictive paths from awe and interpretive bias at the previous wave to interpretive bias and mental health at the next wave. Concurrently, we also incorporated the predictive path from awe at Wave 1 to mental health at Wave 3. The fit indices we used to evaluate the measurement model included the ratio of chi‐square value to degrees of freedom (*χ* ^2^/df), Comparative Fit Index (CFI), Incremental Fit Index (IFI), Goodness of Fit Index (GFI), and Root Mean Square Error of Approximation (RMSEA). We set the acceptable values for CFI, IFI, and GFI at 0.90 or above while considering *χ* ^2^/df and RMSEA satisfactory if they were less than 5 and 0.08, respectively (Fang and Wen 2023).

We chose the Cross‐Lagged Panel Model (CLPM) because it is a statistical technique widely applied in longitudinal research to explore the dynamic relationships between variables over time (Cole and Maxwell 2003). There are several advantages to using the CLPM. First, it allows researchers to examine the predictive effects of one variable on another while controlling for correlations between variables at the same time point and the stability of variables over time. Secondly, the CLPM can capture dynamic relationships between variables. Thirdly, the CLPM can clearly distinguish the sequence of variable measurement and fulfill the premise of causality inference, permitting the inference of causality between variables to a certain extent (Fang and Wen 2023; Lüdtke and Robitzsch 2021). Therefore, the CLPM is a powerful tool for establishing causality among variables, which aligns with the research objectives of the present study, exploring the influence of awe on mental health and the mediating role of interpretive bias between them.

## 3 Results

### 3.1 Common Method Variance

As the data in the current study were self‐reported, it is necessary to consider the possibility of common method bias (CMB). To address this concern, Harman's one‐factor method was used to detect CMB (Podsakoff et al. 2012). We included all the measured items, and the results of principal component analysis showed that the explanatory ratios of the first factor accounted for 26.70%, 29.79%, and 30.12% for the first, second, and third waves respectively, which is less than the criterion of 40%. These results suggested that CMB was not a major issue in this study.

### 3.2 Descriptive Statistics for Variables

Descriptive statistics of the variables were shown in Table 1. To assess whether these variables underwent changes over time, we employed repeated measures analysis of variance and post hoc multiple comparisons with the Bonferroni correction (Bland and Altman 1995).

**TABLE 1:** Descriptive statistics and comparison for variables.

Variables	Wave 1	Wave 2	Wave 3	*F*‐test
Dispositional awe	5.07 ± 0.94^a^	5.04 ± 0.95^a^	5.03 ± 0.93^a^	*F* = 0.61; *p* = 0.546; *η* ^2^ = 0.001
Positive interpretation	3.34 ± 0.48^a^	3.42 ± 0.52^b^	3.48 ± 0.51^c^	*F* = 29.97; *p <* 0.001; *η* ^2^ = 0.05
Negative interpretation	2.73 ± 0.65^a^	2.65 ± 0.71^b^	2.64 ± 0.70^b^	*F* = 10.43; *p <* 0.001; *η* ^2^ = 0.02
Neutral interpretation	3.59 ± 0.41^a^	3.64 ± 0.45^b^	3.69 ± 0.45^c^	*F* = 15.39; *p <* 0.001; *η* ^2^ = 0.03
Life satisfaction	4.10 ± 1.18^a^	4.11 ± 1.23^a^	4.19 ± 1.14^a^	*F* = 2.67; *p* = 0.069; *η* ^2^ = 0.01
Happiness	2.88 ± 0.43^a^	2.85 ± 0.44^a^	2.87 ± 0.57^a^	*F* = 0.82; *p* = 0.442; *η* ^2^ = 0.002
SCL‐90	90.31 ± 65.64^a^	83.95 ± 67.35^b^	87.71 ± 68.17^ab^	*F* = 5.09; *p* = 0.006; *η* ^2^ = 0.01

*Note:* The superscript letters a, b, and c represent post hoc comparisons of variables to determine whether there is a significant difference. Different letters indicate significant differences between different waves.

It was found that negative interpretation bias was the highest while positive interpretation and mental health measures scored lowest in Wave 1, which were then improved in Wave 2 and Wave 3. Additionally, there was minimal change in dispositional awe, life satisfaction, and happiness over time, *ps* > 0.069. These results suggest that cognitive function and mental health were significantly and adversely affected at the onset of loosened pandemic policies.

### 3.3 Pearson Correlations for Variables

Results of Correlative analysis (see Table 2) showed that dispositional awe exhibited a positive correlation with positive interpretation, neutral interpretation, life satisfaction, and happiness. Conversely, it was negatively associated with negative interpretation and SCL‐90. In addition, the correlation between awe and the indicators of mental health was also significant.

**TABLE 2:** Pearson correlations between variables.

	1	2	3	4	5	6	7	8	9	10
1 DW_wave1	1									
2 PI_wave1	0.39***	1								
3 NegI_wave1	−0.13**	−0.20***	1							
4 NeuI_wave1	0.17***	0.58***	0.01	1						
5 LS_wave1	0.29***	0.41***	−0.30***	0.07	1					
6 Hap_wave1	0.30***	0.32***	−0.48***	0.13**	0.56***	1				
7 SCL‐90_wave1	−0.12**	−0.09*	0.57***	−0.04	−0.25***	−0.53***	1			
8 DW_wave2	0.60***	0.36***	−0.21***	0.16***	0.34***	0.31***	−0.18***	1		
9 PI_wave2	0.36***	0.67***	−0.23***	0.37***	0.35***	0.28***	−0.13**	0.44***	1	
10 NegI_wave2	−0.22***	−0.26***	0.74***	−0.05	−0.30***	−0.45***	0.49***	−0.22***	−0.23***	1
11 NeuI_wave2	0.18***	0.37***	−0.01	0.50***	0.03	0.09*	−0.02	0.26***	0.63***	−0.02
12 LS_wave2	0.34***	0.34***	−0.32***	0.04	0.63***	0.53***	−0.29***	0.40***	0.41***	−0.35***
13 Hap_wave2	0.32***	0.29***	−0.37***	0.13**	0.46***	0.64***	−0.43***	0.31***	0.36***	−0.44***
14 SCL‐90_wave2	−0.13***	−0.11*	0.48***	−0.06	−0.24***	−0.48***	0.75***	−0.16***	−0.17***	0.54***
15 DW_wave3	0.64***	0.40***	−0.18***	0.19***	0.36***	0.33***	−0.16***	0.70***	0.52***	−0.23***
16 PI_wave3	0.32***	0.60***	−0.28***	0.37***	0.31***	0.30***	−0.20***	0.42***	0.71***	−0.34***
17 NegI_wave3	−0.23***	−0.27***	0.67***	−0.08	−0.33***	−0.39***	0.45***	−0.28***	−0.32***	0.80***
18 NeuI_wave3	0.11*	0.28***	−0.06	0.48***	0.03	0.15***	−0.07	0.22***	0.42***	−0.11*
19 LS_wave3	0.34***	0.38***	−0.28***	0.09*	0.63***	0.53***	−0.26**	0.37***	0.42***	−0.34***
20 Hap_wave3	0.31***	0.34***	−0.43***	0.11*	0.55***	0.64***	−0.48***	0.36***	0.41***	−0.52***
21 SCL‐90_wave3	−0.17***	−0.16***	0.46***	−0.12**	−0.25***	−0.47***	0.75***	−0.20***	−0.23***	0.53***

	11	12	13	14	15	16	17	18	19	20	21
1 DW_wave1											
2 PI_wave1											
3 NegI_wave1											
4 NeuI_wave1											
5 LS_wave1											
6 Hap_wave1											
7 SCL‐90_wave1											
8 DW_wave2											
9 PI_wave2											
10 NegI_wave2											
11 NeuI_wave2	1										
12 LS_wave2	0.07	1									
13 Hap_wave2	0.15***	0.62***	1								
14 SCL‐90_wave2	−0.11*	−0.31***	−0.52***	1							
15 DW_wave3	0.32***	0.39***	0.36***	−0.18***	1						
16 PI_wave3	0.47***	0.34***	0.36***	−0.23***	0.49***	1					
17 NegI_wave3	−0.11**	−0.34***	−0.40***	0.46***	−0.26***	−0.31***	1				
18 NeuI_wave3	0.66***	0.06	0.19***	−0.17***	0.27***	0.61***	−0.10*	1			
19 LS_wave3	0.08	0.70***	0.55***	−0.26***	0.44***	0.39***	−0.34***	0.07	1		
20 Hap_wave3	0.15***	0.59***	0.67***	−0.50***	0.43***	0.44***	−0.55***	0.21***	0.68***	1	
21 SCL‐90_wave3	−0.16***	−0.31***	−0.49***	0.79***	−0.22***	−0.28***	0.53***	−0.23***	−0.28***	−0.58***	1

*Note:* *p < 0.05, **p < 0.01, ***p < 0.001.

Abbreviations: DW, dispositional awe; Hap, happiness; LS, life satisfaction; NegI, negative interpretation; NeuI, neutral interpretation; PI, positive interpretation.

### 3.4 The Longitudinal Model of Dispositional Awe, Interpretation Bias, and Positive Mental Function

**FIGURE 1:**
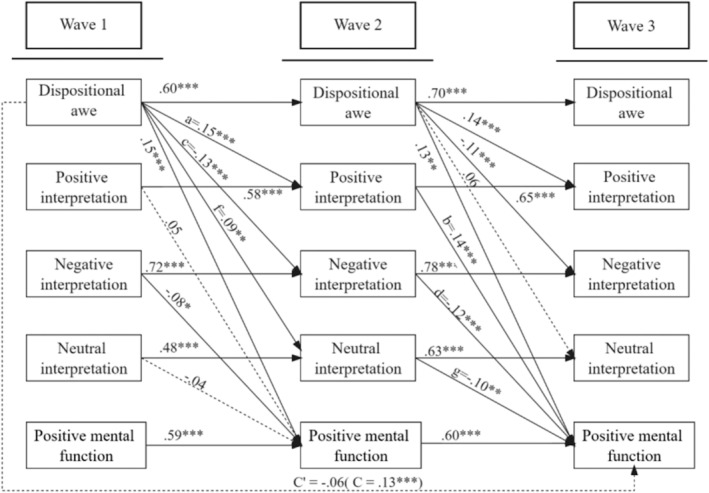
Cross‐lagged panel mediation model for the effect of dispositional awe on positive mental function. All path coefficients were standardized, and dashed lines denoted insignificant paths. The effects of all controlled variables on the outcome variable in Wave 3 were insignificant, *ps* > 0.055, except for gratitude (*β* = 0.09, *p* = 0.008, 95% CI [0.02, 0.16]). Residuals were not shown.

A cross‐lagged structural equation model (SEM) and a parametric bootstrap procedure with 5000 replications were used to calculate 95% bias‐corrected CIs for the indirect effects by the parameters and standard errors (Wang and Zhang 2020). We included dispositional awe as an input variable, positive, negative, and neutral interpretation bias as the mediators, and positive mental function as the outcome variable. Mental health was an explicit variable. We standardized the scores of life satisfaction and happiness, respectively, and then combined the standardized scores to create an index of positive mental function (Liu et al. 2022). In the current study, the input, mediators, and outcome variable were all longitudinal variables, measured from Wave 1 to Wave 3. Meanwhile, we controlled for the effects of gender, age, education level, area, the number of times to contact the virus, the severity, and gratitude on the outcome variable in Wave 3. The model fitting is satisfactory, *χ* ^2^/df = 705.34/173 = 4.08, *p* < 0.001, CFI = 0.91, IFI = 0.91, GFI = 0.90, RMSEM = 0.08. The cross‐lagged panel model was shown in Figure 1.

Results indicated that dispositional awe in Wave 1 and Wave 2 significantly predicted positive and negative interpretation bias in Wave 2 and Wave 3, respectively, and similar results were found regarding interpretive bias and positive mental function. The total effect of dispositional awe in Wave 1 significantly predicted positive mental function in Wave 3 (total effect = 0.13, 95% CI = [0.06, 0.21], *p* = 0.001), positive interpretation (*a* = 0.15; 95% CI = [0.08, 0.22], *p <* 0.001), negative interpretation (*c* = −0.13; 95% CI = [−0.19, −0.07], *p* < 0.001), and neutral interpretation (*f* = 0.09; 95% CI = [0.02, 0.16], *p* = 0.006) in Wave 2. Positive interpretation (*b* = 0.14; 95% CI = [0.06, 0.22], *p* = 0.001), negative interpretation (*d* = −0.12; 95% CI = [−0.19, −0.05], *p* = 0.001), and neutral interpretation (*g* = −0.10; 95% CI = [−0.17, −0.03], *p* = 0.008) in Wave 2 were linked with positive mental function in Wave 3. The indirect effect of dispositional awe on positive mental function was significant through the increased positive interpretation (mediation effect = *a* × *b* = 0.02; 95% CI = [0.01, 0.04], *p* < 0.001, *P*_*M*_ = 20%) and the decreased negative interpretation (mediation effect = *c* × *d* = 0.02; 95% CI = [0.01, 0.03], *p <* 0.001, *P*_*M*_ = 13.33%). In addition, dispositional awe could enhance neutral interpretation, which, however, did not strengthen positive mental function (mediation effect = *f* × *g* = −0.01; 95% CI = [−0.02, −0.002], *p* = 0.007). Additionally, a direct path from dispositional awe in Wave 1 to positive mental function in Wave 3 was insignificant, *β* = −0.06, 95% CI = [−0.14, 0.02], *p* = 0.141. These results indicate that dispositional awe gradually alters individuals' interpretation bias (increase positive and reduce negative interpretation), which, in turn, enhances their happiness and life satisfaction following the administration of loosened COVID‐19 policies.

### 3.5 The Longitudinal Model of Dispositional Awe, Interpretation Bias, and Psychosomatic Symptom

**FIGURE 2:**
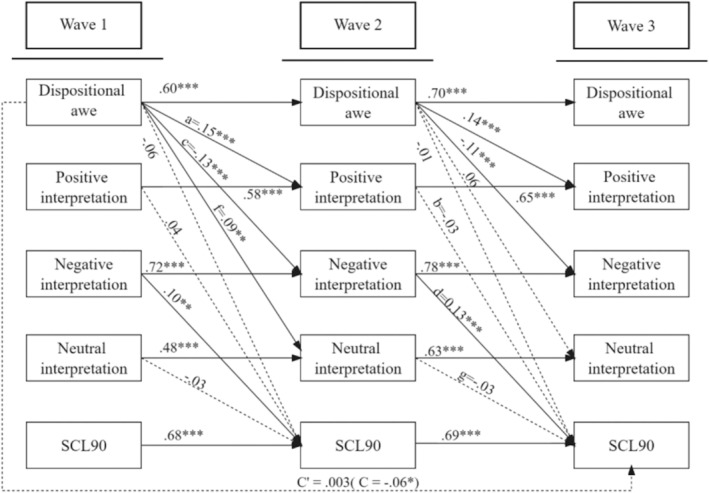
Cross‐lagged panel mediation model for the effect of dispositional awe on psychosomatic symptoms. All path coefficients were standardized, and dashed lines denoted insignificant paths. The effects of all controlled variables on SCL 90 in Wave 3 were insignificant, *ps* > 0.158, except gratitude (*β* = −0.09, *p* = 0.003, 95% CI [−0.15, −0.03]). Residuals were not shown.

Using the same analysis methods, we examined the mediation effects of interpretation bias between dispositional awe and mental symptoms. We included dispositional awe as an input variable, positive, negative, and neutral interpretation bias as the mediators, and SCL‐90 as the outcome variable. The model fitting is satisfactory, *χ* ^2^/df = 697.25/173 = 4.03, *p* < 0.001, CFI = 0.91, IFI = 0.91, GFI = 0.90, RMSEM = 0.08. The cross‐lagged panel model can be observed in Figure 2.

Results indicate that the effect of dispositional awe in Wave 1 had a marginally negative prediction on SCL‐90 in Wave 3 (total effect = −0.06, 95% CI [−0.13, −0.01], *p* = 0.074), and the direct effect was insignificant (direct effect = 0.003, 95% CI [−0.07, 0.08], *p* = 0.952). Meanwhile, negative interpretation in Wave 2 positively associated with SCL‐90 in Wave 3 (d = 0.13; 95% CI = [0.06, 0.21], *p* = 0.001). The indirect effect of awe on SCL‐90 was significantly mediated by the decreased negative interpretation (mediation effect = *c* × *d* = −0.02; 95% CI = [−0.03, −0.01], *p =* 0.001). Neither the mediation effects of positive (mediation effect = *a* × *b* = −0.01; 95% CI [−0.02, 0.01], *p* = 0.319) nor neutral interpretation (mediation effect = *f* × *g* = −0.003; 95% CI [−0.01, 0.002], *p* = 0.203) were significant. These results suggest that dispositional awe can decrease psychosomatic symptoms during COVID‐19 transmission through shaping individuals' interpretation bias.

## 4 Discussion

Prior studies have investigated the influence of awe on mental health and found a positive correlation between awe and well‐being/life satisfaction and a negative correlation between awe and stress, depression, as well as anxiety (Bai et al. 2021; Gordon et al. 2017; Luo et al. 2021; Rankin et al. 2020; Rudd et al. 2012). China has witnessed a sudden surge in COVID‐19 infections during a short period after the administration of loosened pandemic policies. This would impact people's mental health. To explore methods of mitigating the adverse impact, the current study investigated the protective effect of dispositional awe on mental health and the cognitive pathway underlying this association. It is confirmed that dispositional awe contributes to individuals' mental health, which is mediated by interpretation bias.

In terms of the changes in interpretation bias and mental health, based on the results of ANOVA and the autoregressive analysis in the SEM, it was found that interpretation bias and mental health showed both stability and changes across the 2 months following the loosened COVID‐19 policies. Initially, negative interpretation bias was highest, while positive and neutral biases were lowest as the pandemic policies loosened. However, over time, there was a shift toward more positive interpretation bias, with a decrease in negative interpretation and an increase in positive and neutral interpretations in the second and third measures. Interpretation bias, as a cognitive tendency, may exhibit consistency over time due to enduring personality traits or habitual thought patterns (Schick et al. 2013). However, it can also be influenced by factors such as emotions or situational changes (Fink‐Lamotte et al. 2020), which may have helped alleviate psychosomatic symptoms experienced by individuals during the pandemic.

It is found that dispositional awe is a protective factor for mental health, which can elevate happiness and life satisfaction and degrade psychosomatic symptoms, for example, depression and anxiety. These results are consistent with previous studies (Bussing et al. 2021; Gordon et al. 2017; Rankin et al. 2020; Rudd et al. 2012; Sturm et al. 2022; Yaden et al. 2017). Qualitative research has demonstrated that experiencing awe in daily life can improve social adjustment and perception of life in depressed individuals (Schneider 2009). Awe is stimulus‐focused and can improve self‐diminishment by exposing individuals to vast stimuli beyond their understanding (Keltner and Haidt 2003), which helps them take notice of something profoundly important (Bai et al. 2017; Bonner and Friedman 2011; Luo et al. 2023). In addition, awe assists individuals in shifting their attention from daily minutiae toward the outside world and promoting openness to experience (Sawada and Nomura 2020) and tolerance for uncertainty (Rankin et al. 2020). These can assist individuals in adopting a more positive attitude toward difficulties and setbacks in life, ultimately promoting post‐traumatic growth (Seidmahmoodi et al. 2011). Our findings further advance previous research on the awe and mental health association by demonstrating that the feeling of awe positively predicts mental health during the period of COVID‐19 transmission.

Another purpose of this study is to examine the mechanisms that underlie the correlation between dispositional awe and mental health. We found that interpretation bias can explain this association. On the one hand, dispositional awe can significantly predict interpretation bias 1 month later. The theory of mood‐congruent bias posits that individuals often tend to process information with a similar emotional valence as their mood (Voelkle et al. 2014). Moreover, the broaden‐and‐build theory of positive emotions also holds that positive emotions can broaden individuals' cognitive resources (Fredrickson 2001). Awe is a positive self‐transcendent emotion (Stellar et al. 2017), associated with esthetic response (Bonner and Friedman 2011; Keltner and Haidt 2003), which allows individuals to appreciate the beauty of the world around them (Buessing et al. 2014; Lovoll and Saether 2022) and to find the life meaningfulness (Zhao et al. 2019). Meanwhile, experiencing the emotion of awe more frequently in daily life, can help people to face ambiguous or difficult situations with greater strength and wisdom (Van Cappellen and Saroglou 2012). These evidences support the current findings that dispositional awe predicts individuals' positive interpretation of ambiguous scenarios and uncertainties.

On the other hand, we also discovered that following 1 month of implementing the loosened COVID‐19 policy, there was a tendency for individuals' interpretations gradually leaned toward positiveness, showcasing a rise in positive interpretation bias and a decline in negative interpretation bias, which can explain the association between dispositional awe and mental health. Specifically, the mediation effects of increased positive interpretation bias and reduced negative interpretation bias were significant for the protective effect of awe on positive mental function, while the mediation effect of reduced negative interpretation bias was significant for the predictive effect of awe on reduced psychosomatic symptoms. It is suggested that dispositional awe can shape individuals' interpretation bias by promoting them to explain ambiguous and uncertain situations in a more positive manner. Interpretation bias, as a significant cognitive factor, plays a crucial role in mental health (Beck 2008). Numerical studies indicate that interpretation bias has robust effects on individuals' positive mental function and psychosomatic symptoms (Beck 2008; Gawęda et al. 2015; Jopling et al. 2021; Mobini et al. 2014; Sonoda 2007; Vinograd et al. 2020). Intervening toward interpretation bias can also effectively reduce individuals' depression and anxiety while enhancing well‐being (Hurley et al. 2018; Podina et al. 2020). Our research uncovers the protective role of dispositional awe in mental health and its underlying mechanisms, providing a novel perspective by investigating how dispositional awe protects people's mental health through the lens of interpretation bias alterations during a period of the COVID‐19 pandemic.

Additionally, we found that neutral interpretation bias did not have a protective role for positive mental function, though neutral interpretation of ambiguous situations can be positively predicted by dispositional awe. Existing research has also found that the regulation effects of neutral interpretation with no cognitive reconstruction are not satisfactory (Wu et al. 2021). This result suggests that by making a positive interpretation of ambiguous situations can we effectively maintain positive mental function, rather than making a neutral interpretation.

The present study has potential theoretical and practical implications. On the one hand, it offers new insights into the correlation between the feeling of awe and mental health. Previous research has predominantly concentrated on investigating the relationship between awe and mental health. However, the exploration of the underlying mechanisms linking awe and mental health is scarce. In the present study, we investigated the predictive effect of dispositional awe on mental health and further explored the mechanisms from the perspective of interpretation bias alteration during the COVID‐19 pandemic. These findings enrich the existing research on the promotion effect of awe on psychological health and enhance our understanding of the positive effects of awe. On the other hand, these findings provide empirical evidence of how individuals can maintain mental health under the circumstances of the COVID‐19 pandemic. Schneider (2009) claims that awe is the ability to stay moved in day‐to‐day life, which can provide individuals with insight and the fortitude to navigate challenging situations. Therefore, cultivating the trait of awe is worth advocating, which can help individuals maintain a positive outlook on the world (Schneider 2009). This also has enlightening inspiration for public management. Previous literature has identified several methods to evoke the experience of awe, including engagement in activities like mountain climbing, appreciating art, immersing oneself in nature, and even learning from adverse events or encountering depression (Anderson et al. 2018; Luo et al. 2021; Schneider 2009).

Several limitations require further attention. First, although we adopted a longitudinal approach to better examine the relationship between variables in the present work, it was still a correlational study from which causal relationships between variables could not be directly inferred. Future research should use a laboratory approach to trigger the state feeling of awe and create ambiguous or uncertain situations to investigate the causal effect of awe on interpretation bias and mental functions directly. Second, due to the unique nature of this policy, the duration of the longitudinal study is relatively short, consisting of only 2 months. Therefore, it is still necessary to investigate the long‐term predictive effect of awe on mental health in future studies, perhaps using other models of public emergency. Third, all the participants in the current study were Chinese, in the first 2 months after China's administration of loosened pandemic policies. Whether the current findings could be generalizable to other cultural/demographic settings remains undetermined. Similarly, our findings were obtained in the context of public health emergency, and whether they are applicable to other public emergency, such as natural disasters or security emergencies, needs further investigation. Finally, all the participants in this study were healthy individuals. Further research also needs to determine whether the findings can be generalized to psychopathological population, such as those with anxiety or depressive disorder. Investigation into these questions may provide further knowledge about how the feeling of awe modulates people's mental health during emergencies, such as COVID‐19 transmission.

## 5 Conclusion

Though the COVID‐19 transmission at the administration of loosened pandemic policies posed a significant threat to people's mental health, dispositional awe serves as an effective buffer for the mental health during the emergency, as evidenced by increased positive mental function and reduced SCL‐90 symptoms in association with dispositional awe. More importantly, dispositional awe plays a protective role in humans' mental health by increasing positive interpretation orientation and decreasing negative interpretation bias. The findings pinpoint that it is essential to cultivate the experience of awe as an important way to maintain one's mental health and positive interpretation style.

## 7 Conflicts of Interest

The authors declare no conflicts of interest.
